# The NF-κB modulated miR-194-5p/IGF1R/PPFIBP axis is crucial for the tumorigenesis of ovarian cancer

**DOI:** 10.7150/jca.40604

**Published:** 2020-03-13

**Authors:** Ru Bai, Kaikai Dou, Yang Wu, Yongjing Ma, Jianmin Sun

**Affiliations:** 1 School of Basic Medical Sciences, Ningxia Medical University, Yinchuan, China; 2 School of Pharmacy, Ningxia Medical University, Yinchuan, China; 3 Department of Gynecological Tumors Surgery, General Hospital of Ningxia Medical University, Yinchuan, China; 4 Division of Translational Cancer Research, Lund Stem Cell Center, Department of Laboratory Medicine, Lund University, Lund, Sweden

**Keywords:** NF-κB, miR-194-5p, IGF1R, PPFIBP, ovarian cancer

## Abstract

miRNAs are involved in the tumorigenesis of various malignancies. In the current study, we found that miR-194-5p expression is downregulated in ovarian cancer tissues, and downregulation of miR-194-5p expression promotes proliferation, invasion and migration of human ovarian cancer cells *in vitro* and ovarian tumor growth in nude mice. We further found that IGF1R and PPFIBP are targets of miR-194-5p, and downregulation of miR-194-5p expression increases IGF1R and PPFIBP expression, resulting in increased proliferation, invasion and migration of ovarian cancer cells. Moreover, we showed that NF-κB can bind to the promoter region of miR-194-5p, and negatively regulate the expression of miR-194-5p in ovarian cancer cells. Taken together, our results suggested a NF-κB modulated miR-194-5p/IGF1R/ PPFIBP axis that is crucial for the tumorigenesis of ovarian cancer, which provides a new insight into the development of ovarian cancer.

## Introduction

Ovarian cancer is one of the main lethal cause of females worldwide 1. Despite of recent progress in treatment such as advanced surgery and chemotherapy, the overall survival rate of ovarian cancer patients was not much improved 2, 3. It is in urgent need to find early diagnosis methods and promising treatment of ovarian cancer.

MicroRNAs (miRNAs) are 18-25 nucleotides long non-coding RNAs which are involved in post-transcriptional regulation of gene expression through regulating the stability of mRNAs and protein translation. They play important roles in various physiological process such as cell proliferation, apoptosis and differentiation 4-6, and dysregulation of miRNA expression contributes to the oncogenesis of cancer. Previous studies demonstrated that miR-183, miR-598, miR-142-5p, miR-34c, miR-375, miR-1307 and others regulate the pathogenesis of ovarian cancer 7-11.

In this study, by examination of miR-194-5p expression in clinical ovarian cancer tumors, we found that the expression of miR-194-5p in tumors is much lower than in the adjacent normal tissues. We further revealed that loss of miR-194-5p expression increases IGF1R and PPFIBP1 expression, leading to increased ovarian cancer cell proliferation, migration and invasion *in vitro* and tumor growth of ovarian cancer *in vivo*.

## Materials and Methods

### Ethics statement

Ovarian cancer tumor tissues and matched adjacent non-tumor tissues were obtained at the Department of Gynecological Tumors Surgery, General Hospital of Ningxia Medical University. The informed consent was approved by all patients or their relatives. All experiments and animal works were approved by Ethics Committees of Ningxia Medical University.

### Cell culture and transfection

Human ovarian cancer cell lines ES-2 and SKOV3, and HEK293T cells were cultured in RPMI- 1640 (Gibco, Grand Island, NY) containing 10% FBS (Hyclone, Logan, USA), 1% penicillin and streptomycin (Beyotime, Shanghai, China). Lipofectamine 2000 (Thermo Scientific, Carlsbad, CA) was used for transfection according to the manufacturer^'^s instructions.

### qRT-PCR

TRIzol (Invitrogen, Carlsbad, USA) were used to purify total RNA from ovarian cancer tissues or cells. cDNA was synthesized from 1 µg of RNA using Moloney murine leukemia virus reverse transcriptase (Promega, Madison, USA), the primers were listed in Table 1. Both the mature miR-194-5p and the endogenous control U6 snRNA were amplified using the transcribed cDNA as template. The qRT-PCR was performed using the SYBR® Premix Ex Taq™ kit (Takara, Dalian, China) and the iQ5 Real-Time PCR Detection System (Bio-Rad, California, USA).

### Lentivirus infection

The lentiviral vector (Lv-hsa-miR-194-5p up, Lv-hsa-miR-194-5p inhibitor) and its corresponding control lentivirus (Lv-up Control, Lv-inhibitor Control) were obtained from GeneChem (Shanghai, China). ES-2 and SKOV3 cells were infected with the lentivirus according to the manufacturer's protocol.

### Plasmids

NF-κB1 cDNA (without the 3'-UTR) was amplified using a cDNA clone as a template followed by insertion into pcDNA3. IGF1R cDNA and PPFIBP1 cDNA were synthesized and inserted into pcDNA3.

The sh-NF-κB1-pSilencer, sh-IGF1R-pSilencer and sh-PPFIBP1-pSilencer vector were generated by annealing the sense and antisense strands of a hairpin RNA followed by insertion between *BamH* I and *Hind* III sites of pSilencer2.1 neo vector (Ambion).

The sense and antisense strands of both wild-type and mutant 3^'^-UTR of the IGF1R or PPFIBP1 gene containing the predicted core binding site for miR-194-5p were synthesized and annealed followed by insertion into the upstream of the reporter gene in pmirGLO vector.

The putative promoter region of miR-194-5p was predicted using algorithms of Promoter Scan (http:// www-bimas.cit.nih.gov/molbio/proscan/), and it was amplified by PCR followed by insertion into the upstream of the reporter gene in the pGL3-basic/ luciferase vector. Subsequently, the luciferase activity were examined using the Dual-Luciferase Reporter Assay system (Promega).

### Proliferation assay

The CCK8 assay (Dojindo, Kumamoto, Japan) was used to evaluate the proliferation of ES-2 and SKOV3 cells. Cells were seeded in 96-well plats with 8000 cells/well. 10µL of CCK8 was added into the medium in 1, 2, 3, 4 and 5 days after infection respectively. The absorbance at a wavelength of 450 nm was detected using the µQuant universal microplate spectrophotometer (Bio-Tek Instruments, Winooski, VT).

### Colony formation assay

ES-2 or SKOV3 cells were seeded at 300 cells/well (in triplicate) in 12-well plates. The colonies with more than 50 cells were stained and counted at day 7 and 14. The rate of colony formation was calculated with the following formula: (number of colonies/number of seeded cells) × 100%.

### Transwell migration and invasion Assays

For transwell migration assay, 1×10^5^ cells were loaded into the upper chamber of each insert (Corning, Cambridge, USA) containing uncoated polycarbonate membrane. For cell invasion assay, the same amount cells were loaded into the upper chamber of each insert pre-coated with 50 μl Matrigel (Clontech, Mountain View, CA). The lower chambers were loaded with 800 μl of medium containing 20% FBS. After incubation for 24h, cells attached to the lower surface were fixed and stained with crystal violet for 15 min. Cell migration or invasion were photographed and measured under a light microscope at ×200 magnification.

### Establishment of ovarian cancer cells expressing miR-194-5p

Virus expressing Lv-hsa-miR-194-5p up, Lv-hsa-miR-194-5p inhibitor or their respective controls infected SKOV3 cells respectively followed by selection with 1µg/ml puromycin for 3weeks.

### Tumor xenograft in mouse

6×10^6^ SKOV3 cells (stable cell line) were resuspended in 100 µL of RPMI 1640 and injected subcutaneously into the left flank of female BALB/c nude mice (n=10/group, 5-6 weeks old). The tumor sizes were measured every 3 days in 28 days. The tumor volume was calculated by length×width^2^× 1/2.

### miRNA targets prediction

The prediction algorithms of TargetScan and PicTar were used to predict the putative downstream mRNA targets of miR-194-5p.

### Dual Luciferase reporter assay

The wild-type or mutant 3'-untranslated region (3' UTR) of IGF1R or PPFIBP was amplified and cloned into pmirGLO vector (Promega). They were co-transfected into HEK 293 cells with miR-19a-3p mimics, inhibitor or their corresponding control. The Dual Luciferase Reporter Assay kit(Promega) was used to examine the luciferase activity in 48 hours after transfection.

### Western blot

Cells were washed with PBS and lysed in RIPA buffer on ice for 30 min. Cell lysates were seperated by SDS-PAGE and transferred into nitrocellulose membranes. Membranes were blocked at room temperature for 2 h, then incubated with primary antibody (Abcam) overnight at 4℃. After washing, the membranes were incubated with HRP conjugated secondary antibody (Tianjin Sier, China) for 1 h at room temperature. Membranes were washed and developed with chemiluminescent solution, the images were acquired and analyzed using LabWorks image acquisition and analysis software (UVP, Upland, CA).

### Chromatin immunoprecipitation assay (ChIP)-PCR

ChIP assay examined the interaction between NF-κB and the promoter of miR-194-5p using Magna ChIP Chromatin Immunoprecipitation Kit (Millipore, Billerica, USA). ES-2 cells were trypsinized and incubated with 1% formaldehyde for 10 min at room temperature. The cells were washed twice with ice-cold PBS, then sonicated to shear DNA to be 100 to 1000bp long followed by electrophoresis to confirm the length of DNA fragments. 100µg chromatin samples were incubated with 1µg anti-NF-κB1 antibody (cat. SRP00225, Tianjin Sier) or anti-mouse IgG antibody(MBL, Japan) overnight at room temperature to precipitate DNA-protein complexes. DNAs were amplified by PCR with the primers flanking the predicted NF-κB1 binding site in the miR-194-5p promoter. Primer sequences were listed in Table 1.

### Electrophoretic mobility shift assay (EMSA)

ES-2 cells were lysed in a lysis buffer (0.3 M sucrose, 60 mM NaCl, 15 mM Tris-Cl pH 8.0, 10 mM EDTA). The biotin-labeled probe was prepared by Biotin 3'End DNA Labeling Kit (Thermo, Pierce, USA). 2 μg of ES-2 cell lysate, 5 mM MgCl, 2.5% Glycerol, 0.05% NP-40, 1 ng/μl of poly (dI·dC) and 4 pmol labeled probes mixed in 20μl binding reaction mixture system and incubated for 20 min at room temperature. After incubation, the mixture was separated by 6% native polyacrylamide gel and transferred into nylon membrane. After transfer, the membrane was immediately cross-linked under UV-light for 5 min. The target bands were detected using chemiluminescence (Thermo, Pierce, USA). The sequences of probes used were listed in Table 1.

### Statistical analysis

The data of at least 3 independent experiments were presented as the mean ± S.D. The data was analyzed by GraphPad Prism software with the two-tailed Student^'^s *t* test. The value of* p* less than 0.05 was considered as significant (* *p*<0.05, ** *p*<0.01).

## Results

### miR-194-5p suppresses the proliferation, migration and invasion of ovarian cancer cells *in vitro*

**Figure 1:**
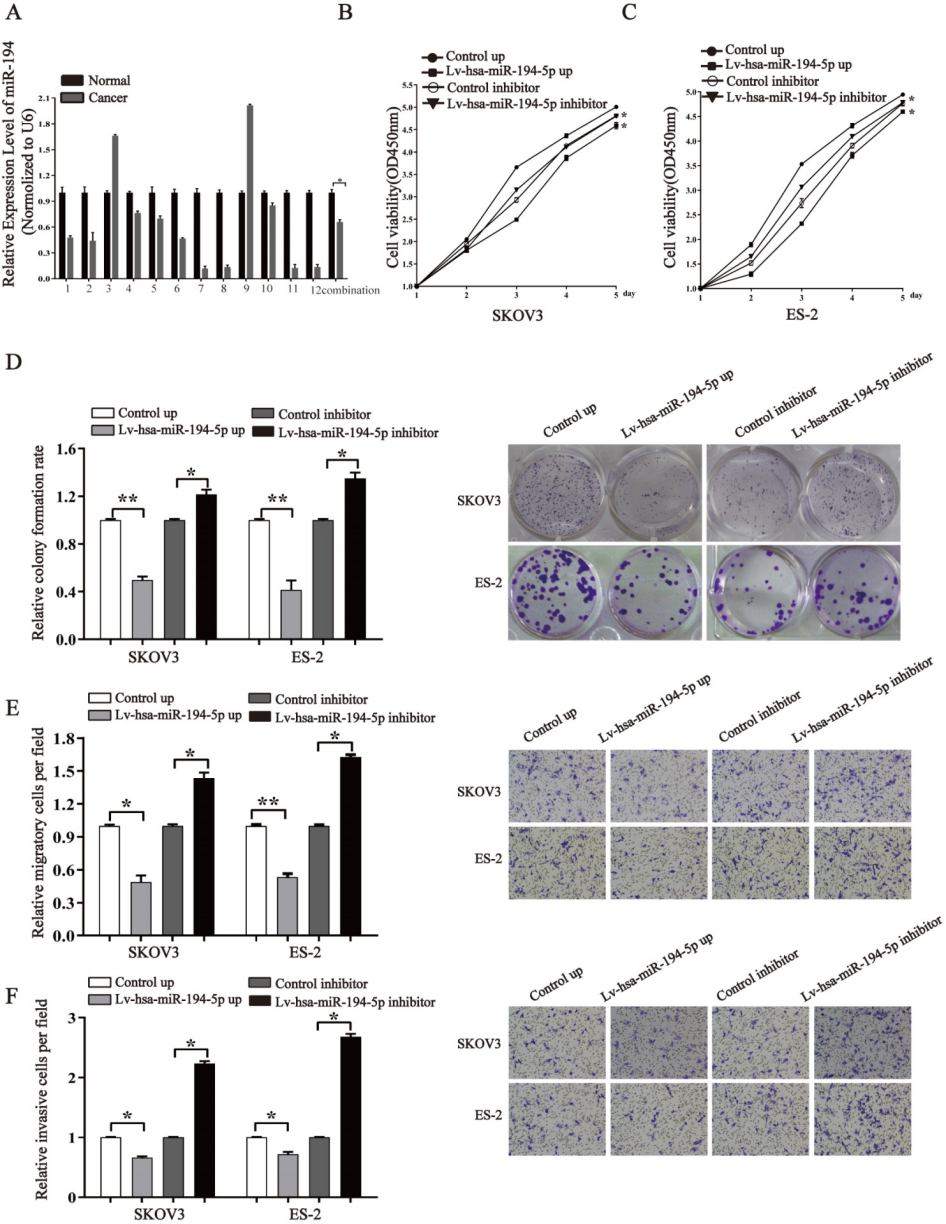
** miR-194-5p is a tumor suppressor in ovarian cancer.** (a) qRT-PCR detected the expression of miR-194-5p in 12 ovarian cancer tissues and their corresponding non-tumor tissues. U6 was used for normalization. (b) and (c) After transfection with Lv-hsa-miR-194-5p up or Lv-hsa-miR-194-5p inhibitor, the proliferation of SKOV3 or ES-2 cells was examined by CCK-8 assay. (d) The colony formation rate was calculated with the following equation: colony formation rate= (number of colonies/ number of planted cells) × 100%. The colony formation rate of cells infected with empty lentiviral vector was defined as 1. (e) and (f) Transwell migration or invasion assay of SKOV3 and ES-2 cells. Cells in three random fields of view at 100x magnification were counted and expressed as the average number of cells per field. The average cell number of its corresponding control group was defined as 1 (*, *p*<0.05, **, *p*<0.01).

To know whether miR-194-5p expression is dysregulated in ovaian cancer, we detected the expression of miR-194-5p in 12 human clinical ovarian cancer tissues using qRT-PCR. The results revealed that the expression of miR-194-5p in ovarian cancer tissues is lower than that in the adjacent non-tumor tissues (Fig. 1a), suggesting the dysregulation of miR-194-5p expression in ovarian cancer and a possible role of miR-194-5p in the oncogenesis of ovarian cancer. To elucidate the function of miR-194-5p in ovarian carcer, miR-194-5p was overexpressed or its expression was knocked down in SKOV3 and ES-2 cells. The CCK-8 assay showed that overexpression of miR-194-5p and knockdown of miR-194-5p expression respectively inhibits and promotes the proliferation of both SKOV3 and ES-2 cells (Fig 1b and 1c). Colony formation assay further revealed that overexpression or knockdown of miR-194-5p respectively decreases or increases the colony formation of both SKOV3 and ES-2 cells (Fig. 1d). In addition, we found that upregulation of miR-194-5p expression can suppress the migration and invasion of both SKOV3 and ES-2 cells, and cell migration and invasion can be promoted by knockdown of miR-194-5p by transwell assay (Fig. 1e and 1f). These results suggested that miR-194-5p inhibits the oncogenesis of ovarian cancer *in vitro*.

### miR-194-5p inhibits the tumor growth of ovarian cancer *in vivo*

**Figure 2:**
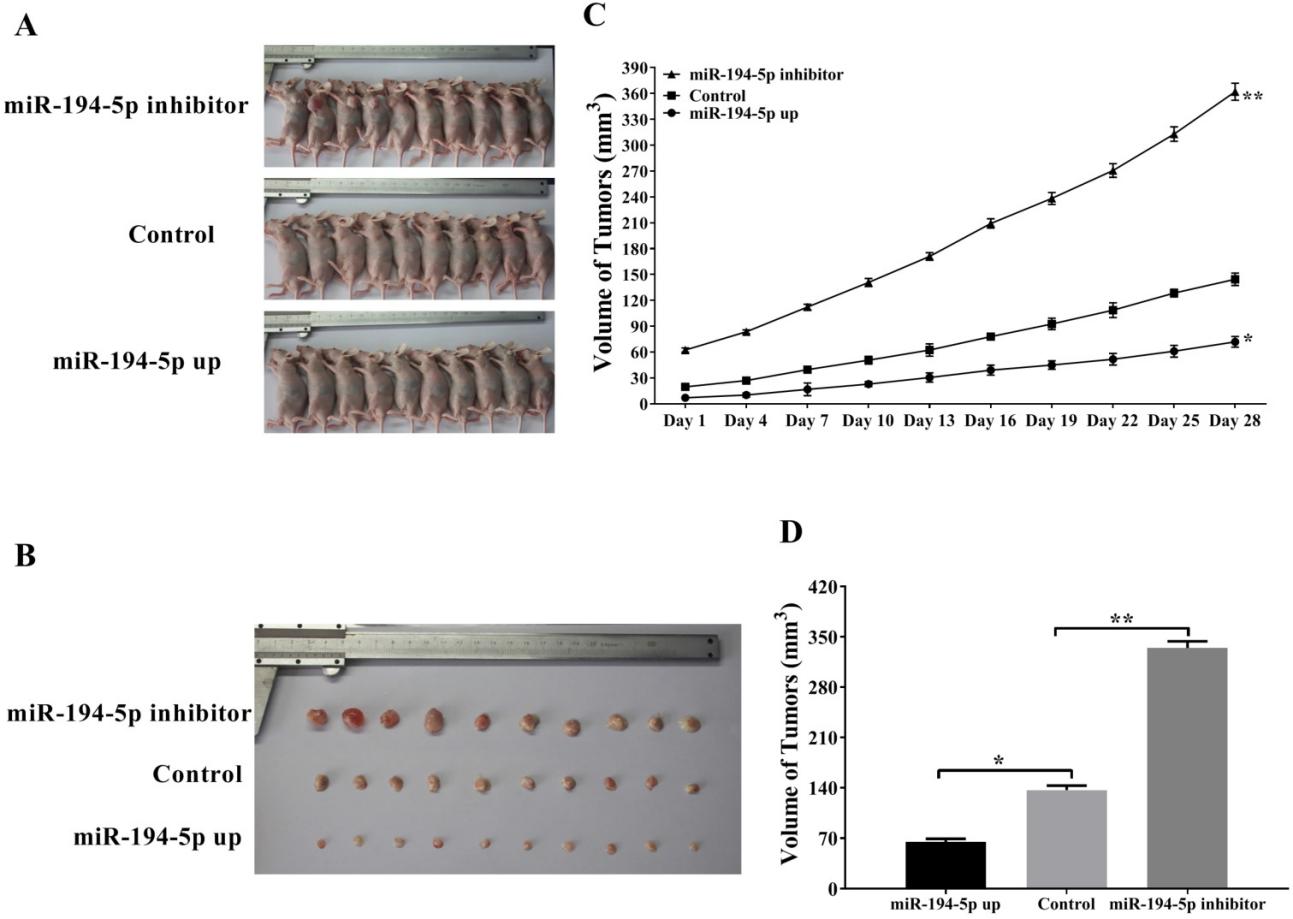
** miR-194-5p suppresses the tumor growth of ovarian cancer in the xenograft model.** (a) SKOV3 cells with miR-194-5p overexpress or knockdown were injected subcutaneously into nude mice. (b) Tumor size was measured after the mice were sacrificed. (c) Tumor size was monitored at different time points. (d) The average size of the tumors was calculated (**, *p*<0.01).

To further explore the role of miR-194-5p in the tumorigenesis of ovarian cancer *in vivo*, SKOV3 cells were injected into the flank of nude mice subcutaneously in order to establish xenograft model (Fig. 2a and 2b). As shown in figure 2c and 2d, tumors overexpressing miR-194-5p grown slower and tumors with miR-194-5p knockdown grown faster than the control, meaning that miR-194-5p inhibits the tumor growth of ovarian cancer *in vivo*.

### Both IGF1R and PPFIBP1 expression are downregulated by miR-194-5p

In order to further know that how miR-194-5p regulates the oncogenesis of ovarian cancer, the potential downstream targets of miR-194-5p were predicted using two bioinformatics databases (TargetScan and PicTar). Among the potential targets, IGF1R and PPFIBP1 were further studies since that they are well-known oncogenes in various malignancies 12, 13.

**Figure 3:**
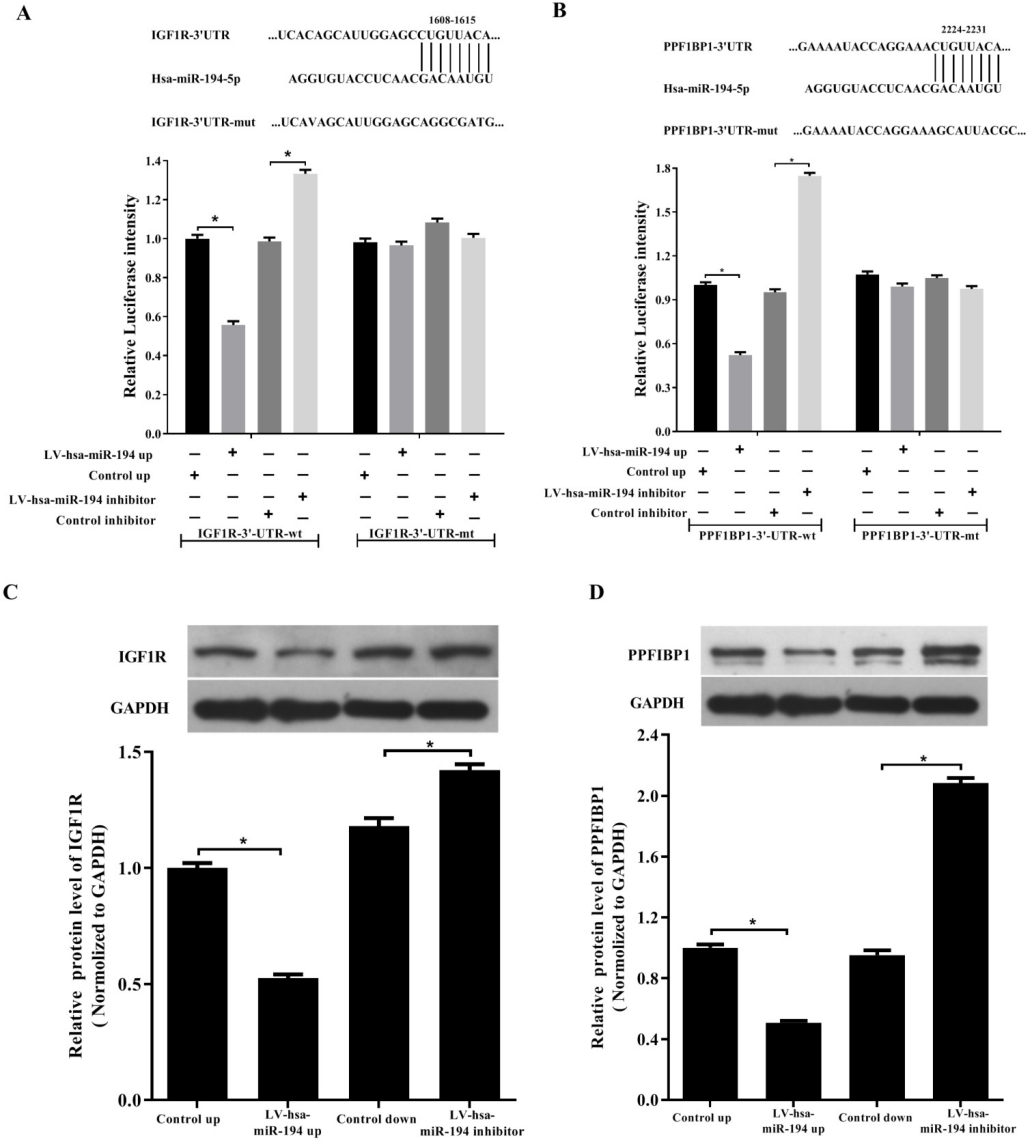
** IGF1R and PPFIBP1 are direct targets of miR-194-5p.** (a) and (b) Predicted miR-194-5p binding sites in the 3'UTR of IGF1R and PPFIBP1. HEK293T cells were transfected with pGL3-WT or pGL3-MT in the presence or absence of Lv-hsa-miR-194-5p up or Lv-hsa-miR-194-5p inhibitor, the luciferase assay examined the luciferase activity of 3'UTR of IGF1R and PPFIBP1 respectively. (c) and (d) HEK293T cells were transfected with Lv-hsa-miR-194-5p up or Lv-hsa-miR-194-5p inhibitor, western blot analysis detected the protein expression of IGF1R and PPFIBP1 respectively. The results are representative of three independent experiments (*, *p*<0.05).

By base-pairing complementation, we found that the 3' untranslated region (UTR) of IGF1R and PPFIBP1 have putative sites that are conserved among species and the two sites are complementary with the seed sequence of miR-194-5p (Fig. 3a and 3b). Dual- luciferase reporter assay revealed that overexpression or knockdown of miR-194-5p respectively reduces or increases the fluorescence intensity in HEK293T cells expressing 3'-UTR of IGF1R or PPFIBP1 but not 3'UTR of IGF1R or PPF1BP1 which contains mutated miR-194-5p binding sites (Fig. 3a, b), meaning that miR-194-5p can directly bind to the 3'-UTR of IGF1R and PPFIBP1, and IGF1R and PPFIBP1 are targets of miR-194-5p in ovarian cancer. Subsequent western blot assay suggested that overexpression or knockdown of miR-194-5p respectively reduces or enhances the expression of IGF1R and PPFIBP1 (Fig. 3c and 3d), which further proved that miR-194-5p negatively regulates IGF1R and PPFIBP1 expression.

### IGF1R promotes ovarian cancer cell proliferation, migration and invasion

**Figure 4:**
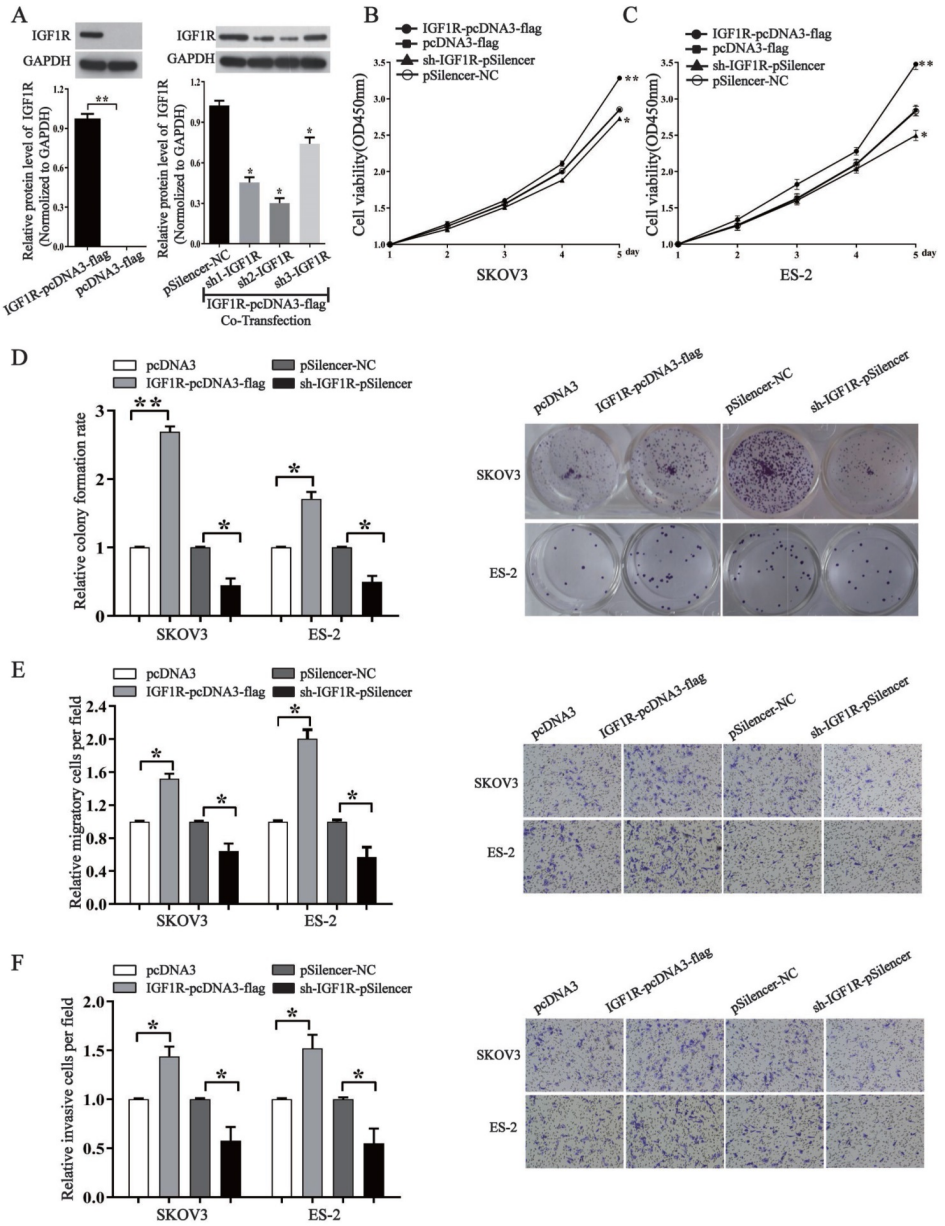
** IGF1R promotes the proliferation, migration and invasion of ovarian cancer cells.** (a) Western blot detected the expression of IGF1R after transfection with IGF1R overexpression or knock down. The results are representative of three independent experiments. (b) and (c) After IGF1R was overexpressed or knocked down, CCK-8 assay examined proliferation of SKOV3 and ES-2 cells respectively. (d) The colony formation rate was calculated with the following equation: colony formation rate= (number of colonies/ number of planted cells) × 100%. The colony formation rate of cells transfected with its control vector was defined as 1. (e) and (f) Transwell migration and invasion assay of SKOV3 and ES-2 cells. Cells in three random fields of view at 100x magnification were counted and expressed as the average number of cells per field. The average cell number of its corresponding control was defined as 1 (*, *p*<0.05, **, *p*<0.01).

In order to know whether miR-194-5p regulates the tumorigenesis of ovarian cancer through IGF1R, we overexpressed or knocked down its expression by an IGF1R overexpression vector or a specific shRNA. Western blotting assays validated the efficiency of pcDNA3-IGF1R or shR-IGF1R (Fig. 4a). By CCK8 and colony formation assays, we found that overexpression of IGF1R increases the proliferation of both SKOV3 and ES-2 cells, and the proliferation can be inhibited by knockdown of IGF1R expression (Fig. 4b, 4c and 4d). Transwell migration and invasion assays further revealed that IGF1R positively regulates ovarian cancer cell migration and invasion of as well (Fig. 4e and 4f), meaning that IGF1R positively regulates the oncogenesis of ovarian cancer.

### PPFIBP1 contributes to the oncogenesis of ovarian cancer

**Figure 5:**
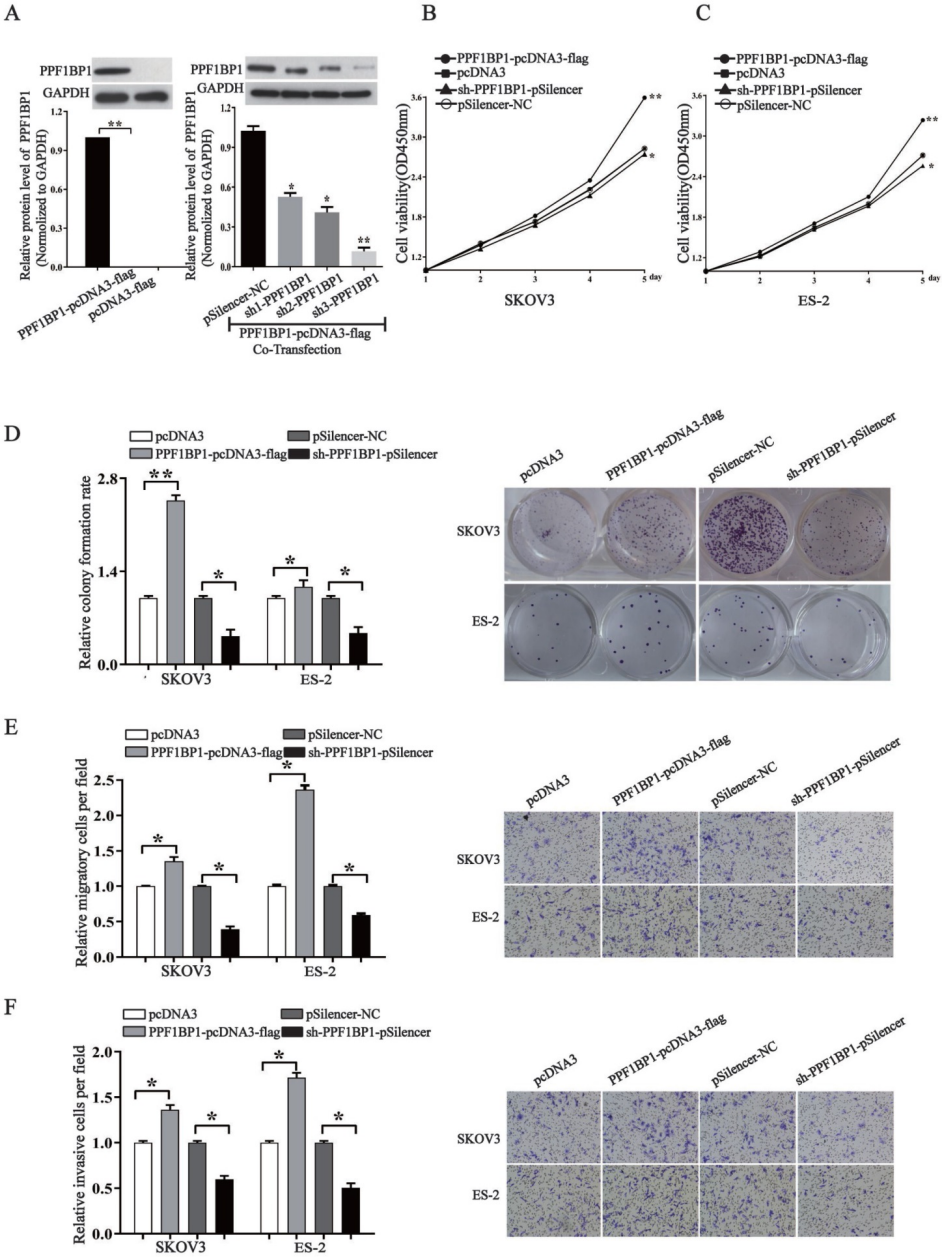
** PPFIBP1 promotes the proliferation, migration and invasion of ovarian cancer cells.** (a) Western blot examined the expression of PPFIBP1. The results are representative of three independent experiments. (b) and (c) CCK-8 assay examined proliferation of SKOV3 and ES-2 cells when PPFIBP1 was overexpressed or knocked down. (d) The colony formation rate was calculated with the following equation: colony formation rate= (number of colonies/ number of planted cells) × 100%. The colony formation rate of cells transfected with its control vector was defined as 1.(e) and (f) Transwell migration and invasion assay of SKOV3 and ES-2 cells. Cells in three random fields of view at 100x magnification were counted and expressed as the average number of cells per field. The average cell number of its corresponding control was defined as 1 (*, *p*<0.05, **,* p*<0.01).

We further elucidated the role of PPFIBP1 in ovarian carcer in order to address that whether miR- 194-5p act as tumor suppressors in ovarian cancer through PPFIBP1 as well. We modified PPFIBP1 expression by overexpression or knockdown using plasmids containing the coding sequence of wild type PPFIBP1 or sh PPFIBP1 (Fig. 5a). Overexpression and knockdown PPFIBP1 respectively increased or inhibited SKOV3 and ES-2 cell proliferation (Fig. 5b and 5c), colony formation (Fig. 5d), migration and invasion (Fig. 5e and 5f), meaning that, similar as IGFR1, PPFIBP1 contributes to the oncogenesis of ovarian cancer.

### NF-κB regulates miR-194-5p, IGF1R and PPFIBP1 expression in ovarian cancer cells

**Figure 6:**
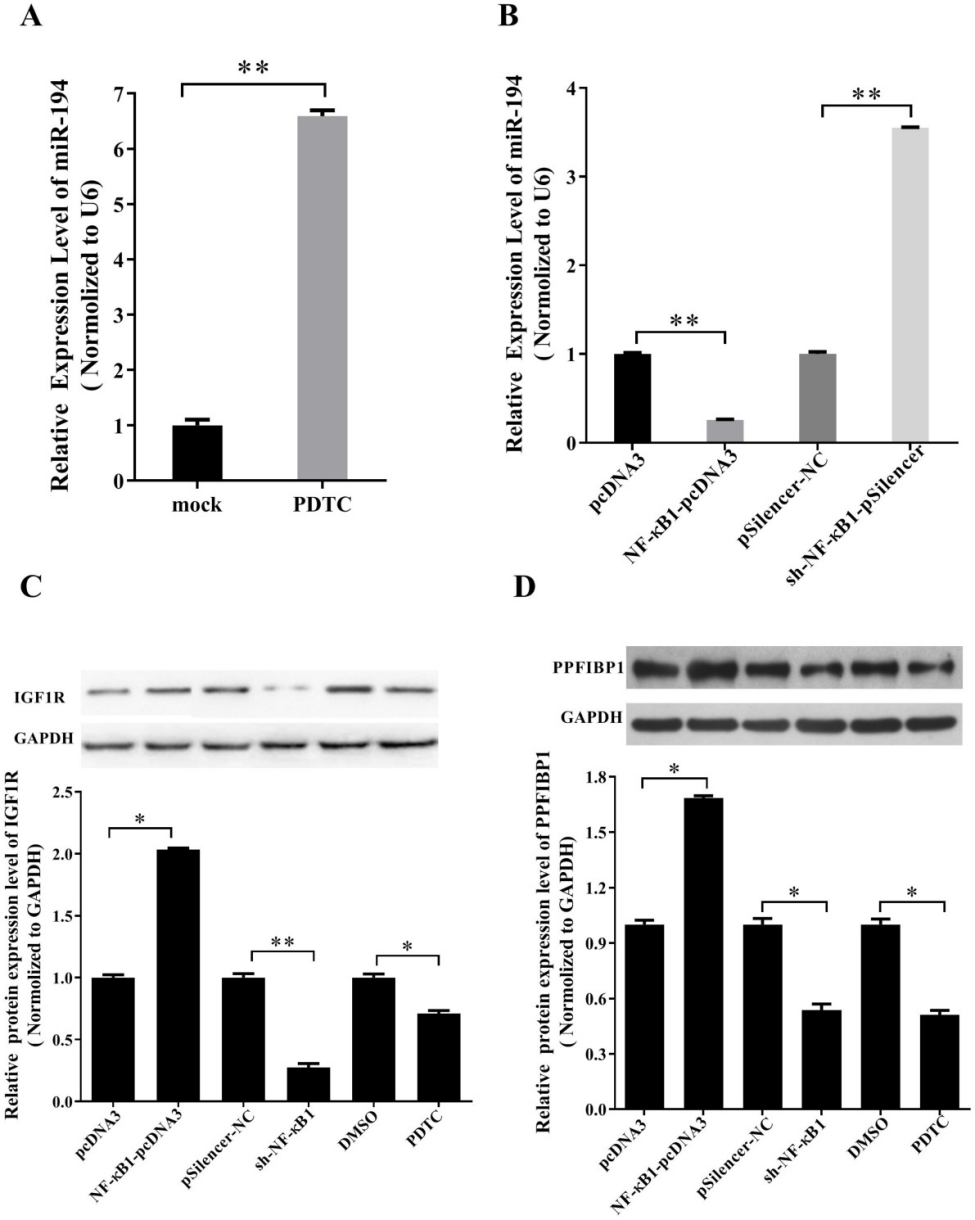
** miR-194-5p, IGF1R and PPFIBP1 expression were inhibited by NF-κB.** (a) The Expression of miR-194-5p in ES-2 cells was determined after pyrrolidine dithiocarbamate (PDTC) treatment. U6 snRNA was used for normalization. (b) qRT-PCR examined the regulation of NF-κB1 on miR-194-5p expression. U6 was used for normalization. (c) and (d) The correlation of protein expression of IGF1R or PPFIBP1 with NF-κB1 expression were analyzed by western blotting(*, *p*<0.05, **, *p*<0.01).

According to our previous study, NF-κB1 can negatively regulate miR-19a expression in ovarian cancer cells 14. To know whether NF-κB1 also regulates miR-194 expression in ovarian cancer, ES-2 cells were treated with NF-κB inhibitor PDTC. qRT-PCR revealed that the expression of miR-194-5p is increased after PDTC treatment (Fig. 6a), indicating that NF-κB1 can inhibit miR-194-5p expression. Overexpression and knockdown of NF-κB1 respectively inhibited or enhanced miR-194-5p expression, which further proved the negative regulation of NF-κB1 on miR-194-5p expression (Fig. 6b).

Since NF-κB1 inhibits miR-194-5p expression and miR-194-5p inhibits IGF1R and PPFIBP1 expression, we further studied that whether NF-κB1 regulates IGF1R and PPFIBP1 expression. Western blot analysis demonstrated that overexpression of NF-κB1 enhances IGF1R and PPFIBP1 expression, while knockdown of NF-κB1 expression and PDTC treatment reduces their expression (Fig. 6c and 6d), meaning that NF-κB1 positively regulates IGF1R and PPFIBP1 expression.

### NF-κB directly binds to the promoter region of miR-194-5p and negatively regulates its expression

**Figure 7:**
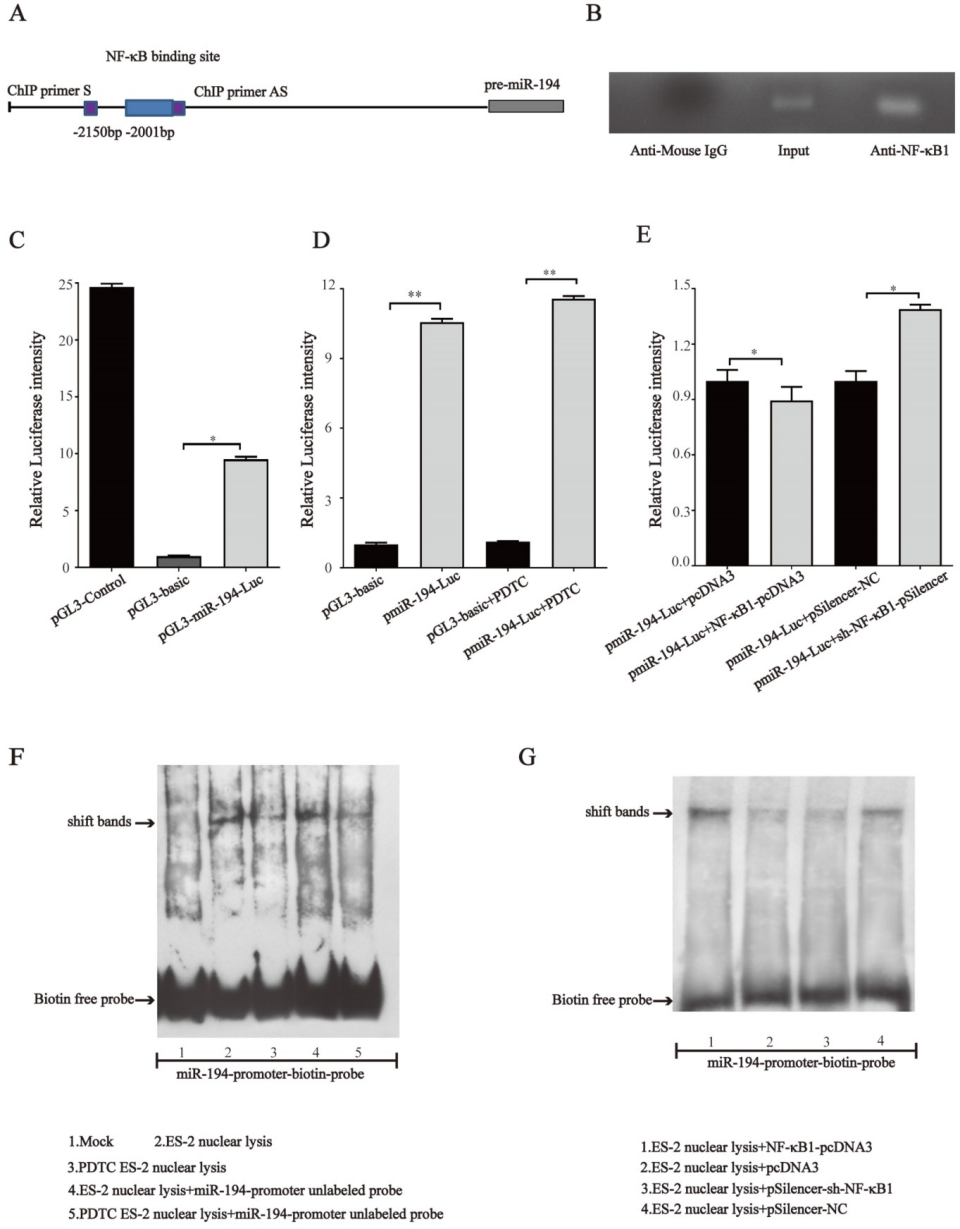
** NF-κB directly binds to miR-194 promoter.** (a) Schematic representation of the miR-194 mRNA and the NF-κB1 binding site. (b) ChIP-PCR analyses of NF-κB binding to the miR-194 promoter using NF-κB1 antibodies. ChIP primers are located at -2001bp ~ -2150bp upstream of the miR-194 gene. (c) The luciferase reporter assay showed that the fragment upstream of the miR-194 gene has strong promoter activity. (d) The luciferase intensity of miR-194 in ES-2 cells after PDTC treatment. (e) The promoter activity of miR-194 in ES-2 cells when NF-κB1 was overexpressed or knocked down. (f) Gel-shift signals by EMSA for the binding site probe incubated with nuclear extracts from ES-2 cells or PDTC treated ES-2 cells. (g) Gel-shift signals by EMSA for the binding site probe incubated with nuclear extracts of ES-2 cells with modified NF-κB1 expression (*, *p*<0.05, **, *p*<0.01).

Since NF-κB1 regulates miR-194-5p expression, we next investigated whether NF-κB regulates miR- 194-5p expression by interaction with the miR-194-5p promoter. We firstly analyzed miR-194-5p promotor by bioinformatics and found a potential binding sites for NF-κB1 at -2001bp upstream of miR-194-5p gene (Fig. 7a). ChIP assay using ES-2 cell lysates and followed PCR amplified miR-194-5p promotor -2001bp ~ -2150bp upstream of the miR-194-5p gene (Fig. 7b), indicating that NF-κB1 can directly interact with the miR-194 promoter. The luciferase reporter assay in ES-2 cells revealed that a DNA fragment upstream of miR-194-5p has promoter activity (Fig. 7c) and PDTC treatment could increase its activity (Fig. 7d), while overexpression or knockdown of NF-κB1 respectively inhibited or enhanced the promotor activity (Fig. 7e), which further confirmed that NF-κB1 inhibits miR-194-5p transcription by interaction with miR-194-5p promotor. EMSA assay revealed a strong gel-shift signal when NF-κB1 binding site probe was added in the ES-2 nuclear extracts and PDTC decreased the amount of binding product (Fig. 7f). Moreover, overexpression or knockdown of NF-κB1 respectively increased or decreased the amount of the binding product (Fig. 7g). These results suggested that NF-κB1 directly binds to specific sites of miR-194-5p promoter in ovarian cancer cells, which suppresses miR-194-5p expression.

## Discussion

MicroRNAs (miRNAs) are a class of short non-coding RNAs that can target mRNAs to regulate the stability and translation of mRNAs, and numerous reports have revealed that the dysregulation of miRNAs plays an important role in tumorigenesis of ovarian cancer.

In the current study, we observed that miR-194-5p is downregulated in ovarian cancer tissues, suggesting a possible role of miR-194-5p in the oncogenesis of ovarian cancer. Our further study revealed that miR-194-5p could prohibit the proliferation, migration and invasion of ovarian cancer cell *in vitro* and ovarian tumor growth *in vivo*, suggesting that miR-194-5p may act as a tumor suppressor in ovarian cancer. Ovarian cancer is not the only cancer whose oncogenesis can be regulated by miR-194-5p, the potential tumor suppressive role of miR-194 has also been demonstrated in other tumors as well. For example, miRNA-194 suppresses gastric cancer cell migration, invasion and epithelial- mesenchymal transition (EMT) by downregulating FoxM1. In human osteosarcoma cell lines, miR-194 reduces cell proliferation, migration and promotes apoptosis by direct inhibition of CDH2 expression. For the patients with advanced colorectal adenoma after polypectomy, miR-194 acts as a promising biomarker that can predict prognosis in adenoma recurrence 15-17. Our current work further expands the current knowledge about the biological functions of miR-194 in the oncogenesis of ovarian cancer.

The insulin-like growth factor I receptor (IGF1R) is highly expressed in various malignancies and the function of IGF1R was demonstrated as an anti- apoptotic factor by facilitating cell survival 12. It has been reported that upregulation of IGF1R expression is correlated with poor outcome of non-small lung cancer 18. PPFIBP1 is also named as liprin β1, it is a 105kDa protein which belongs to leukocyte common antigen-related (LAR) transmembrane tyrosine phosphatase-interacting proteins (liprin) family. PPFIBP1 was identified as a new target for metastasis-associated protein S100A4 which promotes invasiveness and metastasis of primary tumor 13. The expression of PPFIBP1 protein was found to be increased in melanoma cell lines and it might be involved in the regulation of cell migration by interaction with Kank1 19. Overexpressed PPFIBP1 in lymphatic vasculature regulates lymphatic vessel integrity 20. PPFIBP1 also contributes to breast tumor cell motility *in vitro* 19-21. In this study, we found that both IGF1R and PPFIBP1 expression can be inhibited by miR-194-5p in ovarian cancer, and downregulation of miR-194-5p promotes the oncogenesis of ovarian cancer by increased expression of both IGF1R and PPFIBP1. The results revealed the pathway that how miR-194-5p regulates the oncogenesis of ovarian cancer.

Nuclear factor-kappaB (NF-κB) is a widely expressed transcription factor that regulates immunity, inflammation and tumorigenesis 22. Many studies demonstrated that constitutively activated NF-κB plays a vital role in the progression of ovarian cancer 23, 24. The transcription factor NF-κB also plays a role in ovarian cancer development through regulating crucial miRNAs. According to previous reports, NF-κB1, c-Rel, and ELK1 contribute to repressed miR-134 expression by direct binding to promoter region of miR-134 in paclitaxel-resistant human ovarian cancer 25. In addition, expression of miRNAs such as miR-130a and miR-503-5p can be regulated by NF-κB in ovarian cancer 26-28. Our previous study also demonstrated that NF-κB directly targets miR-19a-3p promoter to negatively regulate its expression in ovarian cancer cells 14. In this study, we demonstrated that NF-κB could negatively regulate the transcription of miR-194-5p, which subsequently regulated the expression of IGF1R and PPFIBP1.

Taken together, our data revealed a NF-κB/ miR-194-5p/IGF1R/PPFIBP1 axis in the oncogenesis of ovarian cancer, NF-κB binds directly to miR-194-5p promoter, leading to the downregulation of miR-194- 5p expression, which in turn facilitates the expression of IGF1R and PPFIBP1, and thereby promotes the oncogenesis of ovarian cancer. These results expand our understanding of the NF-κB-mediated regulation network of ovarian cancer.

## Figures and Tables

**Table 1:** The Primers and Oligonucleotides Used in qRT-PCR, CHIP, EMSA and Vector Constructions

Name	Sequence (5' →3')
miR-194-5p RT	GTCGTATCCAGTGCAGGGTCCGAGGTGCACTGGATACGACTCCACATGG
miR-194-5p Forward	TGCGGTGTAACAGCAACTC
U6 RT	GTCGTATCCAGTGCAGGGTCCGAGGTGCACTGGATACGACAAAATATGG
U6 Forward	TGCGGGTGCTCGCTTCGGCAGC
Reverse	CCAGTGCAGGGTCCGAGGT
IGF1R -3'-UTR-Top	AAACTAGCGGCCGCTAGTCCTCACAGCATTGGAGCCTGTTACAGTGCAAGACATGT
IGF1R -3'-UTR-Bot	CTAGACATGTCTTGCACTGTAACAGGCTCCAATGCTGTGAGGACTAGCGGCCGCTAGTTT
IGF1R-3'-UTR-mut- Top	AAACTAGCGGCCGCTAGTCCTCACAGCATTGGAGCAGGCGATGGTGCAAGACATGT
IGF1R-3'-UTR-mut- Bot	CTAGACATGTCTTGCACCATCGCCTGCTCCAATGCTGTGAGGACTAGCGGCCGCTAGTTT
IGF1R -shR-Top	GATCCTCTGTCCCTGTCCTTCCCTGCTCGAGCAGGGAAGGACAGGGACAGATTTTTGA
IGF1R - shR-Bot	AGCTTCAAAAATCTGTCCCTGTCCTTCCCTGCTCGAGCAGGGAAGGACAGGGACAGAG
PPFIBP1-3'-UTR-Top	AAACTAGCGGCCGCTAGTACCAGGAAACTGTTACAGACGCCAT
PPFIBP1-3'-UTR-Bot	CTAGATGGCGTCTGTAACAGTTTCCTGGTACTAGCGGCCGCTAGTTT
PPFIBP1-3'-UTR-mut-Top	AAACTAGCGGCCGCTAGTACCAGGAAAGCATACGCGACGCCAT
PPFIBP1-3'-UTR-mut-Bot	CTAGATGGCGTCGCGTATGCTTTCCTGGTACTAGCGGCCGCTAGTTT
PPFIBP1 - shR-Top	GATCCGCTGATTCAGGAGATCAATGCTCGAGCATTGATCTCCTGAATCAGCTTTTTGA
PPFIBP1 - shR-Bot	AGCTTCAAAAAGCTGATTCAGGAGATCAATGCTCGAGCATTGATCTCCTGAATCAGCG
NF-κB1 -S	CGGAATTCGCCACCAGAATGGCAGAAGATGATC
NF-κB1 -AS	TGTCACTCGAGGCAATTTTGCCTTCTAGAGGTC
NF-κB1- shR-Top	GATCCCGCCTGAACAAATGTTTCATTTGGTCAAGAGCCAAATGAAACATTTGTTCAGGCTTTTTTGGAAA
NF-κB 1- shR-Bot	AGCTTTTCCAAAAAAGCCTGAACAAATGTTTCATTTGGCTCTTGACCAAATGAAACATTTGTTCAGGCGG
miR-194-promoter-S	CGGGGTACCCCACCAAGTTTAGTCAGA
miR-194-promoter-AS	CCGGAATTCCTTGGCTAACTGCAACCTC
miR-194-ChIP-S	GCCGGGCGCGGTGGTTC
miR-194-ChIP-AS	CCACTATGCCTGGCTGAT
GAPDH-ChIP-S	TACTAGCGGTTTTACGGGCG
GAPDH-ChIP-AS	TCGAACAGGAGGAGCAGAGAGCGA
miR-194p-biotin- probe-Top	GACCTACATGAAGAAACCCCATCTCTACTAAATATACAAAAATCAGCCAGGCATAGTGG
miR-194p-biotin- probe-Bot	CCACTATGCCTGGCTGATTTTTGTATATTTAGTAGAGATGGGGTTTCTTCATGTAGGTC
